# Psychometric review of the perceived stress scale under CFA and Rasch models in Lima, Peru

**DOI:** 10.3389/fpsyg.2023.1160466

**Published:** 2023-05-23

**Authors:** Alicia Boluarte-Carbajal, Martín Salazar-Conde, Sandra Alata Vasquez, Angel Zegarra-López

**Affiliations:** ^1^Facultad de Ciencias de la Salud, Escuela de Psicología, Universidad Cesar Vallejo, Lima, Peru; ^2^Facultad de Psicología, Universidad de Lima, Lima, Peru

**Keywords:** stress, perceived stress, validity, reliability, Rasch model

## Abstract

Stress is a public health disease that is increasing rapidly in the population worldwide, so it is necessary to take measures for detection and evaluation, through short scales. The purpose of the study was to analyze the psychometric properties of the Perceived Stress Scale (PSS) in a sample made up of 752 people with an age range of 18 to 62 years (M = 30.18, DE = 10.175), of whom 44% (331) were women and 56% (421) men, from Lima, Peru. The results, by means of confirmatory factor analysis and the Rasch model, confirmed the global adjustment of a 12-item (PSS-12) version with the presence of two orthogonal factors independent of each other, and also demonstrated the metric equivalence according to gender and adequate internal consistency. These results allow us to recommend the use of the PSS-12 in the Peruvian population for the measurement of stress.

## Introduction

Stress is a problem that affects a large number of people to a greater or lesser degree, accentuated by the impact of the state of health emergency that is being experienced internationally. Studies carried out in 130 countries as a result of the pandemic show an increase of up to 72% in mental health care for conditions such as anxiety, depression, withdrawal symptoms, delirium, stress and others (Organizacion Mundial de la Salud, 2020). In 2021, after a year of pandemic, depression and anxiety disorders have been exacerbated by 25%; demand affected by the serious neglect of the health systems in charge, due to budgetary and administrative implications and shortcomings in information and research, producing a global crisis in mental health (Organización Panamericana de la Salud, 2022). All this reflects the large footprint left by the pandemic caused by the new coronavirus, with the urgent need to generate changes in favor of the population.

From a conceptual perspective, stress can be understood as a physiological response of the organism to an intimidating or demanding organism to an intimidating or demanding scenario (Barrio et al., 2006) that occurs when the person is overwhelmed by situations under which he/she cannot exercise control and is confronted with new situations (Martin, 2007). The COVID-19 pandemic has produced serious consequences in the mental health of the population, regardless of the age group to which they belong (Palomino-Oré and Huarcaya-Victoria, 2020), in addition to changes in the new study and work modalities, which demanded a greater load of perceived stress (Estrada Araoz et al., 2021; Sandoval-Reyes et al., 2021).

The “Global Perceived Stress Scale” (PSS-14) created by Cohen et al. (1983) is considered one of the most recognized and widely used instruments to measure stress (Trujillo and González-Cabrera, 2007; Pedrero-Pérez et al., 2015; Sun et al., 2019), with the purpose of measuring the degree to which life experiences are considered stressful, the original version has 14 items, 7 written positively and 7 negatively. The same author later proposed a 10-item version (Cohen and Williamson, 1988) with 2 defined factors, found by Principal Component Analysis (PCA). However, the estimation of factors through PCA means ignoring the measurement error, focusing only on the total variance, the result of which does not reflect the underlying theory of the measured construct (Lloret-Segura et al., 2014).

The PSS has been used in different countries and translated into several languages, mainly in a clinical application setting (Jasis and Guendelman, 1993; López et al., 1995). In Spain, Remor and Carrobles (2001) carried out the first translation of the scale into European Spanish, demonstrating concurrent validity with global stress and anxiety. They later reported the concurrent validity of the PSS-10 with anxiety, internal consistency and test–retest reliability (Remor, 2006).

In the analysis of the internal structure with CFA, few studies report the type of estimator, being the maximum likelihood (ML) used by various authors (Barbosa-Leiker et al., 2013; Guzmán-Yacaman and Reyes-Bossio, 2018; Larzabal-Fernandez and Ramos-Noboa, 2019) as well as least squares (Brito Ortíz et al., 2019). Although the analysis methodology requires specific criteria according to the measurement scale, Li (2016), Dominguez-Lara et al. (2022), and Juárez-García et al. (2021) recommend the least squares estimator diagonally weighted (WLSMV) for demonstrating its effectiveness and suitability for the analysis of variables on an ordinal scale. Regarding dimensionality, the research by Yokokura et al. (2017) in Brazil (PSS-14 and PSS10), Dao-Tran et al. (2017) in Vietnam (PSS-10), Baik et al. (2019) in the United States (PSS-10), Sun et al. (2019) in China (PSS-10), Reyna et al. (2019) in Argentina (EEP-14), Brito Ortíz et al. (2019) in Mexico (EEP-14), Campo-Arias et al. (2020) in Colombia (PSS-10) report the presence of two dimensions, with items written directly and inversely. In Iran, Maroufizadeh et al. (2018) used the 10-item version in infertile women, confirming the two-dimensional structure and its diagnostic accuracy in women. Likewise, Dominguez-Lara et al. (2022) in Peru ruled out unidimensionality and found that the version of 14 items (PSS-14) and 10 items (PSS-10) with two dimensions is acceptable.

Research in which the scale with 14 items was used, such as Benítez et al. (2013) in Venezuela; Larzabal-Fernandez and Ramos-Noboa (2019) in Ecuador and Huang et al. (2020) in China showed a better fit after removing item 12. Likewise, a low interfactorial correlation (r = 0.12; Huang et al., 2020) in PPS-14 when removing item 12.

Other studies propose a bifactor model (Reyna et al., 2019), this solution being widely accepted when there is a domain structure that determines the presence of a general factor with adequate adjustment indices (Rodriguez et al., 2016). Rasch analyses of the PSS have been previously carried out in different contexts. In New Zealand, Medvedev et al. (2019) used an unrestricted Rasch model and found that the PSS-10 could not be treated as a unidimensional scale due to the strong relationships between item residuals in a sample of university students. For this reason, the authors tested each subscale independently and demonstrated that both subscales had strong reliability (r_p_ = 0.80), with excellent coverage, targeting a 98% of the sample, and strong fit to the model. Nielsen et al. (2016) also examined the PSS-10 in a Danish population study. Their results were similar to Medvedev et al. (2019) in the sense that the PSS-10 did not fit the unidimensional Rash model; nevertheless, further Mokken and Rash analyses demonstrated that each subscale could be treated as unidimensional independently, with a significative better fit and strong reliable measures. Finally, Ribeiro-Santiago et al. also found that a two-dimensional structure had a better fit rather than the unidimensional proposal for the PSS-14 in Aboriginal (2014) and Australian (2020) populations. In summary, all previous Rasch studies found that the whole PSS do not fit a unidimensional structure which leads to unordered thresholds and low reliability. So far there is no study where the Rasch multidimensional model has been applied, being adequate in cases where unidimensionality is not met.

Part of the analysis of the internal structure is the invariance of the measurement, whose procedure demonstrates the equivalence and principle of equity of a test (Byrne, 2008). Some studies reported invariance according to gender in the PSS-10 (Barbosa-Leiker et al., 2013; Juárez-García et al., 2021); Another study verified the invariance in Spanish-speaking and English-speaking Hispanic American groups in the United States (Baik et al., 2019), also, a recent study reported the invariance according to gender and occupational status (workers and students) in both versions (Juárez-García et al., 2021), demonstrating that the construct has the same meaning for people regardless of the group to which they belong.

Regarding reliability, studies report internal consistency mostly with Cronbach’s Alpha. In the PSS-10, Medvedev et al. (2019) reports an alpha of 0.88 as a unifactorial structure, while other authors show values fluctuating between 0.656 and 0.810 are shown for global measures (Dao-Tran et al., 2017; Maroufizadeh et al., 2018; Medvedev et al., 2019; Reyna et al., 2019; Sun et al., 2019) and for multidimensional measures, the positive factor ranges from 0.72 to 0.865, while the negative factor ranges from 0.72 to 0.754 (Yokokura et al., 2017; Huang et al., 2020). In addition, Reyna et al. (2019) reports values between ω 0.657 to ω 0.798 of Omega for both global and multidimensional measures, in the PSS_10 and PSS-14 version. Other procedures are also observed to obtain reliability, through the test–retest technique with intervals of 1 month r = 0.43 (Dao-Tran et al., 2017), 1-week positive factor r = 0.820 and negative factor 0.993 for each factor, as well as the ICC = 0.954 (Sun et al., 2019).

The advancement of psychometric research allows combining models, which derive from Item Response Theory (IRT) through Rasch analysis. This last methodological perspective oriented to the construction of measures in the social sciences includes properties that resemble measures in the physical sciences (Bond et al., 2021). The Rasch model considers the latent construct as a continuum in which items and persons can be ordered according to location parameters on a common scale (Wright, 1993). Such ordering and parameters allow estimating the probability with which a person will select a specific response to an item (Andrich and Marais, 2019). Unlike models based on confirmatory factor analysis, where one expects to find a model that best fits a data set, the Rasch model establishes a prescriptive framework to which both individuals and items must conform to ensure a satisfactory calibration of the instrument and an accurate estimation of the level of the latent trait (Lamprianou, 2020).

The little evidence of the psychometric behavior of the scale in our country increases the problem regarding the measurement of stress, in Peru only 2 studies were carried out in specific samples of university students (Guzmán-Yacaman and Reyes-Bossio, 2018) and nurses (Dominguez-Lara et al., 2022), but, there is no study in the general Peruvian population.

Therefore, the present study responds to the need to increase the psychometric evidence of the PSS-13 version (Guzmán-Yacaman and Reyes-Bossio, 2018) and PSS-10 (Cohen et al., 1983) in Metropolitan Lima testing different models with confirmatory factor analysis and Rasch modeling, as well as checking the evidence of equity with respect to gender and finally internal consistency.

## Materials and methods

### Study design

#### Participants

The total sample was 752 people with an age range of 18 to 62 years (= 30.18, SD = 10.175), of which 44% (331) were female and 56% (421) male, 40.4% (304) worked and had family responsibilities, while 26.6% (200) studied and worked, and 23.1% (174) only studied and had family responsibilities. The sample size met the criteria to perform the psychometric analyzes (Lloret-Segura et al., 2014). The sampling method was non-probabilistic, intentional, using the snowball technique or tracking by links as a strategy (Johnson, 2014), being able to access the sample through personal contacts and social networks, which allowed to gradually increase the sample size (Baltar and Gorjup, 2014).

#### Research instruments

The Perceived Stress Scale (PSS), developed by Cohen et al. (1983), was used. This instrument provides a global measure of perceived stress in the last month, that is, the degree to which various life situations are interpreted as stressful. It consists of 14 items, of which items 4, 5, 6, 7, 9, 10, and 13 are positive statements and items 1, 2, 3, 8, 11, 12, and 14 have negative directionality. The scaling is ordinal (0 = never, 1 = almost never, 2 = sometimes, 3 = often, 4 = very often) and the total score was obtained by inverting the positive items. The present study uses the version adapted in Peru by Guzmán-Yacaman and Reyes-Bossio (2018), composed of 13 items.

#### Ethical aspects

During all the research stages, ethical aspects were taken into consideration, such as the Declaration of Helsinki, guaranteeing at all times the protection of personal data, confidentiality and voluntary participation, as well as the four basic principles of bioethics: autonomy, beneficence, non-maleficence, and the principle of justice (Sánchez, 2009).

#### Procedure

Due to current healthcare circumstances, data collection was carried out virtually, making use of information and communication techniques, for which a Google form was edited, containing information on the objective of the study, informed consent, sociodemographic data and the measurement instrument.

#### Data analysis

Descriptive and psychometric analyses were performed using the RStudio interface, version 4.2.0 (2020). Descriptive statistics was used to obtain the mean (M), standard deviation (SD), kurtosis coefficients (g1) and asymmetry (g2); to analyze the distribution of scores (Forero et al., 2009; Pérez and Medrano, 2010), they observed the magnitudes of the corrected homogeneity index (IHC) with values higher than 0.30, indicating that the reagent consistently contributes with the measurement (Kline, 2005); communalities (h2) were observed with values higher than 0.40, interpreting that the items have a common content (Lloret-Segura et al., 2014; Lloret et al., 2017). Such processing was executed using the psych library (Revelle, 2023).

The analysis of the internal structure of the test was performed with a matrix of polychoric correlations, considering the ordinal nature of the items (Jöreskog, 1994; Finney and DiStefano, 2013; Hoffmann and Stover, 2013; Domínguez, 2014) and as estimation method we used Weighted Least Squares with mean and variance adjusted (WLSMV) in line with the need to correct for nonlinearity and normality (DiStefano and Morgan, 2014; Brown, 2015). For the model evaluation phase, fit indexes such as Chi-square over degrees of freedom χ^2^/gL, less than 3 (Hair et al., 2009) were used. In addition, Comparative Fit Index (CFI) greater than 0.95 (Lai, 2020), Tucker-Lewis Indices (TLI) greater than 0.90 (Xia and Yang, 2019), being these two incremental indices. The literature points out that it is optimal if the values are higher than 0.95 (Hu and Bentler, 1999; Kline, 2011; Brown, 2015; Escobedo Portillo et al., 2016). Likewise, the Root mean square error of approximation (RMSEA) with its confidence intervals (RMSEA CI 90%) lower than 0.08 (Hu and Bentler, 1998) and Standardized root mean square residual (SRMR), with a magnitude lower than 0.05 (Bentler and Bonet, 1980; Lai, 2020), for both cases. Finally, the Weighted Root Mean Residual (WRMR) should be close to 1 (Ching-Yun, 2002; Gelabert et al., 2011). All these fit indexes were obtained using the syntax associated with the Lavaan package (Rosseel, 2012).

However, as fit indices tend to favor hierarchical models, specific bifactor indices were used (Dominguez-lara and Rodriguez, 2017). In this way, to assess whether a general factor is possible, the following statistics were used: the explained common variance (ECV), which is the variance they have in common attributed to the general factor (Ten Berge and Sočan, 2004; Sijtsma, 2009) that should be greater than 0.60 (Reise et al., 2013), besides the percentage of uncontaminated correlations PUC, which is that proportion that is exempt from the disturbance caused by multidimensionality (Rodriguez et al., 2016) and finally, to consider the strength of the general factor, the hierarchical omega (ωHG) was calculated, which is the variance attributed to the general factor (Zinbarg et al., 2006), considering adequate values higher or equal to 0.70 (Rodriguez et al., 2016).

Subsequently, the evidence of fairness was evaluated by means of multigroup confirmatory factor analysis in relation to gender (Byrne, 2008), considering three levels of restriction to test the model obtained: (1) Configural invariance (without restrictions), (2) Metric invariance (first phase that restricts the factorial loadings), (3) Strong invariance (restricts the loadings and Intercepts), and (4) Strict invariance (restricts the loadings, Intercepts and residuals), observing the changes in the ΔCFI ≤ 0.01, ΔRMSEA ≤ 0.015, ΔSRMR ≤ 0.030 (Chen, 2007). 01, ΔRMSEA ≤ 0.015, ΔSRMR ≤ 0.030 (Chen, 2007). and in case this exceeds that value, it would signal that the instrument does not possess the quality that both groups interpret the scale in the same way. Consequently, the scores would not have the same interpretation. These analyses were performed using the semTools package.

A Rasch analysis was employed to evaluate the measures derived from applying the PSS-10 (Cohen et al., 1983) and PSS-13 (Guzmán-Yacaman and Reyes-Bossio, 2018).

In principle, given the polytomous nature of the PSS items and the evidence previously found in favor of a potential unidimensional structure, we proceeded to fit the unidimensional models for both versions of the instrument, specifically with the Rating Scale Model (RSM; Andrich, 1978) given that the items share a common Likert scale, which imposes a restriction on the homogeneous structure of thresholds for all items of the scale. Likewise, multidimensional models were fitted for both scales, based on the Mixed-Coefficients Multinominal Logit Model (MCMLM; Adams and Wu, 2007). The overall fit of the models was tested using various statistics that allow testing the covariance between residuals of pairs of items, to identify potential violations in the assumption of local independence. Among them, the MAD_aQ3_ statistic that corresponds to the average of the absolute values of the Q3 statistic in its version adjusted (i.e., centered) to Q3; so that values close to 0 would indicate no relationship among the residuals between pairs of items and support for local independence.

In addition, the MADRESIDCOV statistic was also estimated as an approximation to the covariance of residuals between pairs of items. These results are accompanied with the square root of the SRMR standardized residuals and the square root of the average of correlations between SRMSR squared residuals (Maydeu-Olivares, 2013). Model comparison was developed considering multiple measures such as AIC, BIC, AICc, and AIC3, and the log-likelihood ratio for nested models. All location measures for items were estimated using the Marginal Maximum Likelihood (MML) algorithm, and person measures were estimated using the Expected *a Posteriori* (EAP) method. The reliability of person separation (R_p_) is reported, as well as comparisons between estimated measures for items among the different methods considering the adjustment of each one of them to the Rasch models proposed through the Outfit and Infit indicators, with expected values between 0.60 and 1.40 as they are rating scales (Wright and Linacre, 1994).

Finally, reliability was analyzed using the internal consistency method, taking into account the model with the highest theoretical and empirical coherence, with the omega coefficient for multidimensional scales and with an ordinal categorical nature (Flora, 2020) obtained through the EFAtools package (Steiner and Grieder, 2020). However, the ordinal alpha was estimated, whose interpretation should be taken with caution in case it does not comply with the tau equivalence principle (Ventura-León, 2018), which states that the factor loadings are statistically equal (Dunn et al., 2014).

## Results

Initially, a descriptive analysis of the items was carried out (Table 1), checking that there is no ceiling or floor effect, variability and a marked absence of acquiescence. The mean indicated that the response tendency is between alternatives 2 and 3, with an SD between 1 and 1.5. Likewise, the asymmetry (g1) and kurtosis (g2) coefficients did not exceed ±1.5.

**Table 1:** Descriptive analysis of the items of the PSS-13 scale.

Factors	Ítems	%					*M*	*SD*	*g_1_*	*g_2_*	*CHI*	*h^2^*	Matrix of polychoric correlations											
		0	1	2	3	4							1	2	3	8	11	13	4	5	6	7	9	10
F1 (Distress)	1	7.71	18.48	48.40	17.15	8.24	2.00	1.00	0.04	−0.07	0.69	0.61	1	-	-	-	-	-	-	-	-	-	-	-
	2	13.70	31.12	31.91	17.02	6.25	1.71	1.09	0.25	−0.60	0.63	0.48	0.55	1	-	-	-	-	-	-	-	-	-	-
	3	6.38	17.42	38.56	25.13	12.50	2.20	1.07	−0.10	−0.49	0.73	0.69	0.70	0.59	1	-	-	-	-	-	-	-	-	-
	8	8.64	20.48	42.15	21.68	7.05	1.98	1.03	−0.05	−0.35	0.26	0.08	0.14	0.17	0.19	1	-	-	-	-	-	-	-	-
	11	4.65	18.22	42.42	23.01	11.70	2.19	1.02	0.02	−0.38	0.72	0.61	0.62	0.50	0.62	0.27	1	-	-	-	-	-	-	-
	13	4.65	19.68	38.83	23.01	13.83	2.22	1.06	0.03	−0.58	0.71	0.59	0.55	0.55	0.61	0.30	0.63	1	-	-	-	-	-	-
F2 (Eustress)	4	9.04	23.94	23.27	24.20	19.55	2.21	1.26	−0.09	−1.09	0.76	0.64	−0.09	0.02	−0.05	−0.15	−0.01	−0.05	1	-	-	-	-	-
	5	10.77	24.87	26.86	21.81	15.69	2.07	1.23	0.03	−1.00	0.76	0.63	0.02	0.01	−0.08	−0.19	0.02	−0.04	0.67	1	-	-	-	-
	6	11.17	25.13	19.81	22.87	21.01	2.17	1.32	−0.07	−1.21	0.85	0.79	−0.04	−0.04	−0.06	−0.18	−0.02	−0.06	0.74	0.75	1	-	-	-
	7	6.52	20.88	35.11	21.28	16.22	2.20	1.14	0.00	−0.76	0.79	0.69	−0.02	0.03	−0.08	−0.14	0.02	−0.01	0.64	0.62	0.73	1	-	-
	9	6.52	23.67	28.06	21.94	19.81	2.25	1.20	−0.03	−1.01	0.80	0.71	−0.06	−0.03	−0.10	−0.20	−0.02	−0.02	0.67	0.62	0.75	0.72	1	-
	10	5.45	21.41	35.64	21.54	15.96	2.21	1.12	0.03	−0.75	0.70	0.52	0.01	0.06	−0.06	−0.23	0.03	0.00	0.53	0.57	0.58	0.66	0.64	1
	12	3.59	24.60	35.24	21.68	14.89	2.20	1.08	0.14	−0.79	0.70	0.53	−0.07	0.01	−0.07	−0.20	0.03	0.00	0.57	0.59	0.64	0.58	0.61	0.55

%, frequency; M, mean; SD, standard deviation; g1, asymmetry; g2, kurtosis; CHI, corrected homogeneity index; h2, communalities.

Regarding the magnitudes of the corrected homogeneity index (CHI), most were above 0.30, except for item 8. In the case of the communalities, almost all met the parameter of being above 0.40 being considered acceptable, showing a good contribution to the overall model; however, item 8 was not acceptable in both communality and CHI. Finally, the correlation of the items denoted that there were no cases of multicollinearity, as they all obtained values below 0.90, respectively. Consequently, it was decided to remove item 8 for further analysis.

### Analysis of the internal structure

For the confirmatory factor analysis, structural models were specified, which are shown in Table 2: correlated factors model, which although offering an acceptable fit, it indicated an interfactor correlation (φ) of −0.041. Accordingly, an uncorrelated factors model was tested, obtaining optimal adjustment indexes, since χ^2^/gL was lower than 3; in addition, CFI higher than 0.95 and TLI higher than 0.90. Likewise, RMSEA obtained an optimal level, being lower than 0.05 and SRMR lower than 0.05, and RMSEA confidence intervals (RMSEA CI 90%) lower than 0.08; and finally, WRMR close to 1.

**Table 2:** Confirmatory factor analysis of the PSS-13 scale.

Models	χ^2^	gL	χ^2^/gL	CFI	TLI	RMSEA	IC 90% RMSEA		SRMR	WRMR
							Inferior	Superior		
Model 1 correlated factors	184.895^***^	53	3.489	0.990	0.987	0.058	0.049	0.067	0.033	1.020
Model 2 orthogonal factors (uncorrelated)	97.010^***^	54	1.796	0.997	0.996	0.033	0.022	0.043	0.037	1.104
Model 3 bifactor	108.619^***^	42	2.586	0.995	0.992	0.046	0.035	0.057	0.022	0.666

^***^Omits the significance associated with the χ^2^, being in all models lower than 0.001.

Regarding the bifactor model, its specific indexes indicated a Common Explained Variance (CEV) of 0.538, hierarchical omega coefficient of the general factor (ωHG) of 0.550 and Percentage of Uncontaminated Correlations (PUC) of 0.53, so the presence of a global factor is rejected, since for it to be possible the CEV must be higher than 0.60 and ωHG higher than 0.70.

### Invariance of measurement

From the model of uncorrelated factors, the measurement invariance in relation to gender was calculated, evaluating step by step the levels of configural invariance, metric or weak invariance, strong or scalar invariance and strict invariance. In agreement with the CFA, the robust WLSMV estimator was used due to the categorical nature of the variables. In this way, the fit of the configural model, considered as the base model on which restrictions are exerted in the subsequent levels, was evaluated (CFI = 0.993, RMSEA = 0.041, SRMR = 0.049), and the findings allowed us to continue with the evaluation of the other levels. Metric invariance, in which loadings are restricted, reported an optimal fit, CFI = 0.992, RMSEA = 0.041, SRMR = 0.049, with minimal changes in CFI < 0.01, Δ RMSEA < 0.015 and ΔSRMR < 0.030 with respect to the configural level, indicating that the items contribute to a similar degree in the measurement of the construct. Consequently, it was possible to reach the next level where the equivalence between intercepts was evaluated (strong level), and the fit was maintained, CFI =0.992, SRMR = 0.06, RMSEA = 0.049. Once again, it is observed that the changes are few, the variable components manage to capture all the means in the variance shared by the items, allowing group comparison (Lee et al., 2015). Finally, the level of strict invariance was analyzed, in which the loadings, intercepts and residuals are restricted, also obtaining acceptable indices, CFI = 0.988, RMSEA = 0.048 and SRMR = 0.052, which would indicate that both the specific variance and the variance of the errors are equivalent among the groups, that is, the scores of this instrument have the same meaning in the groups examined (Table 3).

**Table 3:** Factor invariance analysis by gender of the PSS scale (Women = 331 and Men = 421).

Niveles	χ^2^	Δχ^2^	gl	Δgl	CFI	ΔCFI	RMSEA	ΔRMSEA	SRMR	ΔSRMR	Pr (>Chisq)
Configural	215.402	−	132	−	0.993	−	0.041	−	0.049	−	−
Metric	233.085	17.683	142	10	0.992	−0.001	0.041	0.000	0.049	0.000	*
Strong	245.452	12.367	152	10	0.992	0.000	0.040	−0.001	0.049	0.000	…
Strict	304.540	59.088	164	12	0.988	−0.004	0.048	0.007	0.052	0.003	***

Δχ^2^, Variations of χ^2^; Δgl, Variations of degrees of freedom; ΔCFI, Variations of CFI; ΔRMSEA, Variations of RMSEA; ΔSRMR, Variations of SRMR; …, Δχ is not statistically significant; * Δχ is statistically significant at the 0.05 level, ***Δχ is statistically significant at the 0.001 level.

### Rasch analysis

Table 4 presents the results of the overall fit of the data toward the four proposed Rasch models. Regarding the results in PSS-13, the statistics 𝑀𝐴𝐷_𝑎𝑄3_, 𝑀𝐴𝐷𝑅𝐸𝑆𝐼𝐷𝐶𝑂𝑉, 𝑆𝑅𝑀𝑅 and 𝑆𝑅𝑀𝑆𝑅show that the fit of the multidimensional model has smaller deviations with respect to the local independence assumption by presenting average correlations and covariances closer to zero. A similar pattern is observed in the results of the PSS-10, since there is a moderate correlation among the residuals of the responses to the items in the unidimensional model, which would denote the presence of other factors that are causing these correlations outside the effect of the proposed latent variable. These deviations regarding the local independence are minimized in the multidimensional model. Removing item 8 from both scales identifies a slight improvement in the fit of the multidimensional model; an improvement that is not observed in the unidimensional models.

**Table 4:** Global adjustment of models.

Scale	Model	MAD_aQ3_	MADRESIDCOV	SRMR	SRMSR
PSS-13	RSM	0.38363	0.38189	0.29040	0.30576
	MCMLM	0.09358	0.10047	0.09570	0.07182
PSS-13 (without item 8)	RSM	0.40099	0.39629	0.29949	0.30409
	MCMLM	0.09627	0.06752	0.04222	0.05161
PSS-10	RSM	0.36223	0.33383	0.27098	0.29956
	MCMLM	0.11304	0.10539	0.08475	0.11330
PSS-10 (without item 8)	RSM	0.42539	0.36531	0.29529	0.30201
	MCMLM	0.11856	0.05229	0.03785	0.04527

${\mathrm{MAD}}_{{\mathrm{aQ}}_{3}}$ , Mean Absolute Deviation of the adjusted $Q_{3}$ statistic; MADRESIDCOV, Mean Absolute Deviation of Residual Covariances; SRMR, Standardized Root Mean Square of Residuals; SRMSR, Standardized Root Mean Square Root of Squared Residuals.

Table 5 shows the formal comparison of the models proposed from the Rasch perspective. In general, the comparative measures of fit indicate that the multidimensional models have a higher fit compared to the unidimensional models in both versions of the scale. The test based on the log-likelihood ratio indicates statistically significant differences between the nested models from the unidimensional and multidimensional perspective for PSS-13 𝜒^2^(2) = 2917.204, 𝑝 < 0.001, and for PSS-10 𝜒^2^(2) =2021.814, 𝑝 < 0.001. This result is also identified when considering PSS-13 without item 8 𝜒^2^(2) = 2855.122, 𝑝 < 0.001 and for PSS-10 without item 8 𝜒^2^(2) = 2106.456, 𝑝 < 0.001.

**Table 5:** Comparison of models.

Scale	Model	Log-Ver	Dev	Par	AIC	BIC	AIC3	AICc
PSS-13	RSM	−13969.874	27939.748	17	27973.748	28052.335	27990.748	27974.582
	MCMLM	−12511.272	25022.545	19	25060.545	25148.377	25079.545	25061.583
PSS-13 (without item 8)	RSM	−12766.989	25533.978	16	25565.978	25639.942	25581.978	25566.718
	MCMLM	−11339.428	22678.856	18	22714.856	22798.065	22732.856	22715.789
PSS-10	RSM	−10780.776	21561.552	14	21589.552	21654.271	21603.552	21590.122
	MCMLM	−9769.869	19539.738	16	19571.738	19645.702	19587.738	19572.479
PSS-10 (without item 8)	RSM	−9637.972	19275.945	13	19301.945	19362.040	19314.945	19302.438
	MCMLM	−8584.744	17169.489	15	17199.489	17268.830	17214.489	17200.141

Log-Ver, log likelihood; Dev, Deviance; Pair, Number of parameters; AIC, Akaike Information Criterion; BIC, Bayesian Information Criterion; AIC3, AIC with penalty px3; AICc, corrected AIC.

Table 6 summarizes the measures of item location and their respective fit indicators with regard to the Rasch model. In the one-dimensional model applied to PSS-13, the estimated threshold parameter measures were found in increasing order 𝛿_1_ = −1.292, 𝛿_2_ = −0.389, 𝛿_3_ = 0.730 and 𝛿_4_ = 0.951.

**Table 6:** Measurements and adjustment of the PSS-13 and PSS-10 items.

Item	EPGE-13								EPGE-10							
	RSM				MCMLM				RSM				MCMLM			
	Measure	SE	Outfit	Infit	Measure	SE	Outfit	Infit	Measure	SE	Outfit	Infit	Measure	SE	Outfit	Infit
Ítem 1	−0.065	0.037	0.924	0.924	−0.061	0.046	0.832	0.831	−0.053	0.037	0.786	0.785	−0.054	0.047	0.851	0.855
Ítem 2	0.233	0.038	1.081	1.088	0.406	0.047	1.092	1.114	0.244	0.037	0.958	0.965	0.427	0.048	1.120	1.142
Ítem 3	−0.270	0.037	1.050	1.055	−0.382	0.046	0.855	0.862	−0.257	0.037	0.894	0.897	−0.385	0.047	0.873	0.881
Ítem 8	−0.047	0.037	1.380	1.294	−0.033	0.046	**1.555**	**1.535**	−0.035	0.037	1.194	1.144	−0.026	0.047	**1.576**	**1.568**
Ítem 11	−0.259	0.037	0.886	0.889	−0.365	0.046	0.814	0.816	−0.247	0.037	0.765	0.767	−0.368	0.047	0.830	0.834
Ítem 13	−0.287	0.037	0.984	0.989	−0.409	0.046	0.878	0.885	−0.275	0.037	0.851	0.854	−0.413	0.047	0.897	0.907
Ítem 4	−0.283	0.037	1.080	1.085	−0.425	0.048	1.107	1.119	-	-	-	-	-	-	-	-
Ítem 5	−0.136	0.037	1.016	1.031	−0.172	0.048	1.018	1.046	-	-	-	-	-	-	-	-
Ítem 6	−0.244	0.037	1.134	1.148	−0.357	0.048	0.931	0.981	−0.232	0.037	1.396	**1.406**	−0.366	0.049	1.077	1.129
Ítem 7	−0.268	0.037	0.832	0.837	−0.399	0.048	0.871	0.860	−0.256	0.037	1.000	1.002	−0.409	0.049	0.843	0.818
Ítem 9	−0.319	0.037	0.969	0.969	−0.487	0.048	0.882	0.907	−0.307	0.037	1.157	1.161	−0.500	0.049	0.865	0.886
Ítem 10	−0.282	0.037	0.868	0.870	−0.422	0.048	1.117	1.092	−0.269	0.037	1.006	1.006	−0.433	0.049	1.058	1.037
Ítem 12	−0.267	0.037	0.845	0.836	−0.397	0.048	1.086	1.040	-	-	-	-	-	-	-	-

SE, Standard error; Outfit, outlier-sensitive fit; Infit, information-weighted fit.

Bold values denote an infit or outfit above the suggested range for rating scales.

No item showed mismatch with the Rasch model and its measures achieved a high degree of reliability 𝑅_𝑝_ = 0.799. A similar result was identified for the multidimensional model with regard to the increasing ordering of the threshold parameters 𝛿_1_ = −2.265, 𝛿_2_ = −0.676, 𝛿_3_ = 1.030 and 𝛿_4_ = 1.911, and the high reliability 𝑅 𝑝_𝑑𝑖𝑠𝑡𝑟é𝑠_ = 0.823; 𝑅 𝑝_eu𝑠𝑡𝑟é𝑠_ = 0.903; however, item 8 presented a mismatch. The correlation between the two dimensions was 𝑟 = −0.180. Regarding PSS-10, the one-dimensional model presented an increasing ordering of the threshold parameters 𝛿_1_ = −1.240, 𝛿_2_ = −0.472, 𝛿_3_ = 0.766 and 𝛿_4_ = 0.946. Item 6 presented a slight mismatch, and the measures reached an acceptable reliability 𝑅_𝑝_ = 0.718. The multidimensional model presented similar characteristics, with an increasing ordering of thresholds 𝛿_1_ = −2.280, 𝛿_2_ = −0.763, 𝛿_3_ = 1.086 and 𝛿_4_ = 1.956 a high reliability for both dimensions 𝑅 𝑝 _𝑑𝑖𝑠𝑡𝑟é𝑠_ = 0.827, 𝑅 𝑝 _eu𝑠𝑡𝑟é𝑠_ = 0.854, and a correlation of 𝑟 = −0.102. In addition, item 8 presented a significant mismatch with respect to the Rasch model.

### Reliability by internal consistency

From the factor loadings and the matrix of polychoric correlations, the omega coefficient was calculated, being 0.859 for the factor of negative items and 0.919 for positive items, considered adequate. Likewise, the ordinal alpha coefficient was found to be 0.879 and 0.926 for each factor, respectively.

## Discussion

Stress is a risk factor associated with the presence of cardiovascular disease. It is considered as the second cause of death and the third cause of disability (León Regal et al., 2018; Jerez Ríos and Madero-Cabib, 2021). Therefore, any action to measure and control stress would help prevent the years of life lost due to disability, because of the burden of illness generated by the sequelae of chronic disease associated with stress (Fernández de Larrea-Baz et al., 2015).

The Perceived Stress Scale (Cohen et al., 1983) was chosen in order to evaluate its factorial structure and to identify to what extent the data correspond to a theoretical structure consistent with the sociocultural context in the Peruvian setting, using the Rasch model together with models based on CFA in order to provide more information on the psychometric properties of the scale and the possibility of constructing measures based on stress as a latent construct. Originally, authors Cohen et al. (1983) reported the scale with a unidimensional structure, demonstrating evidence of convergent validity and reliability. The dimensionality of the PSS was contrasted years later by Cohen and Williamson (1988), using principal component analysis with varimax rotation, a methodology that was not appropriate for analyzing the internal structure of the test (Martínez and Sepúlveda, 2012). In that study, the results reported the presence of two factors, one with items written with a positive directionality and the other with items written in the opposite direction. On the basis of these findings, the authors affirm a two-dimensional structure apparently due to the statistical result of the factor analysis. In other words, the two-dimensional structure, which the authors found in light of the existing literature, is based on the positive/negative wording of the items, currently called method effect, identified by the authors Cohen and Williamson (1988). This theoretical gap in the dimensionality of the PSS gave rise to the various denominations to the factors: eustress and distress (Guzmán-Yacaman and Reyes-Bossio, 2018), perception and coping (Reyna et al., 2019), perceived helplessness and perceived self-efficacy (Roberti et al., 2006; Maroufizadeh et al., 2018), control and loss of control (Pedrero-Pérez et al., 2015), coping ability and perceived stress (Puentes Martínez et al., 2019).

Being the method effect one of the problems presented by the PSS due to the negative phrasing of the items, many psychometric studies report distorted results as a consequence of reverse and/or negative items (Tomás et al., 2012; Tomas et al., 2013; Rodrigo-Comino et al., 2019). In this sense, the presence of systematic error is assumed, a product of the instrument design, which could affect the internal validity of the study (Barraza et al., 2019), theoretically it was replaced by item 13.

### Item analysis

Analyzing the findings, initially descriptive, item 8 had little contribution to the measurement of the construct; a similar situation was found by Yokokura et al. (2017) who also identified a low factor loading, a reason that motivated the elimination of the item from the psychometric analyses. Observing the phrasing of item 8: “*In the last month, how often did you realize that you could not do all the things you should do?”* may have been interpreted differently, since, in the item construction process, the use of generic words implying “universality” such as the word “*all*” is discouraged (Moreno et al., 2006; Muñiz and Fonseca-Pedrero, 2019).

### Validity evidence based on the internal structure

Through the CFA, different models were structurally tested and considering the ordinal nature of the data, the weighted least squares estimator with adjusted mean and variance (WLSMV) was used as the selection criterion; also in other studies (Yokokura et al., 2017; Brito Ortíz et al., 2019; Reyna et al., 2019), being the model of uncorrelated factors the one that showed the best fit indices with an interfactorial correlation of r = −0.041. This finding is consistent with the results of Moral de la Rubia and Cázares De León (2014) whose relationship between the factors was not significant (r = −0.15); similar case occurred in the adaptation of Guzmán-Yacaman and Reyes-Bossio (2018), Pedrero-Pérez et al. (2015), and Puentes Martínez et al. (2019). These findings confirm the presence of two clearly differentiated factors in the measurement of PSS, which is evidence that both variables are independent in measuring stress and could be used as subscales (Baik et al., 2019). However, the literature evidences studies that confirm the existence of 2 clearly related factors (Remor, 2006; Pedrero-Pérez et al. (2015); Yokokura et al., 2017; Reyna et al., 2019; Huang et al., 2020) concluding that both the factor named “***eustress*** and ***distress***” by Guzmán-Yacaman and Reyes-Bossio (2018) are negatively correlated (r = −0.41), representing to be dependent variables for the measurement of global stress, which contrasts with the findings encountered (r = −0.041).

In response to this, the bifactor model was tested to explain the presence of a single factor with stress being the general factor and two specific factors created by positive and negative wording. Although the fit indices for the bifactor model were adequate, there is a tendency for these results to favor the Common Explained Variance (ECV), hierarchical omega coefficient of the General factor (ωHG) Percentage of Uncontaminated Correlations (PUC; Dominguez-lara and Rodriguez, 2017). The findings encountered in the specific indices ruled out the presence of a general factor representing both dimensions, a similar situation reported by Dominguez-Lara et al. (2022). However, this specific analysis identifying the presence of a general factor in a bifactor model is not evidenced in the study of Reyna et al. (2019), being necessary to report this analysis to justify the influence of a general factor explaining the higher variability compared to those specific of the PSS (Reise et al., 2013).

### Measurement invariance

The evaluation of measurement invariance with the gender variable was performed, finding at the configural, metric, strong and strict levels adequate fit indices (CFI < 0.01, Δ RMSEA < 0.015 and ΔSRMR < 0.030). This finding permitted to identify that the measurement of stress with the PSS is invariant in men and women, evidence that confirms the gender similarity hypothesis proposed by Hyde (2005).

### Rasch model

In the same way, it was identified in the versions of PSS-10 and PSS-13 that item 8 presented a mismatch with regard to the Rasch model. Such a mismatch would imply that individuals with higher levels of the latent trait are selecting alternatives that would indicate a low level of stress, and that individuals with low levels of the latent trait are selecting alternatives that indicate a high level of stress, both phenomena with very low probability given the postulates of the model. In the studies by Santiago et al. (2019) and Nielsen et al. (2016), the item with the highest mismatch was item 4.

However, in both studies the English version of the PSS was used; whereas, in the present work a Spanish translation is used that considers cultural aspects which could explain why we identified different mismatch patterns with what was observed in the literature (Guzmán-Yacaman and Reyes-Bossio, 2018). After removing item 8 from the scale, it is observed that no remaining item mismatches with the model. In addition, an increase in the reliability of person separation is observed, suggesting that the appropriate ordering of individuals according to their level in the latent trait is more accurate after removing this item. The reliability identified in our study for the dimensions employing the MCMLM model are higher than those observed by the studies of Santiago et al. (2019) and Nielsen et al. (2016) who employed a differential cluster modeling approach. It is important to recognize that in the studies by Santiago et al. (2019) and Nielsen et al. (2016) they employ as an approximation the unconstrained Rash model, also called Partial Credit Model (PCM), while in this study the Rating Scale Model (RSM) is used since all items share a common Likert scale.

After analyzing the response patterns observed in the PSS scale and its fit with respect to the Rasch model, it was identified that the unidimensional model did not show the assumption of local independence. This limitation is overcome by using multidimensional modeling, which indicates that the two-factor structure allows for more specificity in explaining the covariance among item responses, given the observed fit indicators. This tendency was also identified in previous studies that employed the Rasch model for the analysis of PSS. Specifically, Santiago et al. (2019) identified in a principal component analysis of the model residuals that the residuals of the responses to the distress and eustress items on the PSS-14 loaded on a principal factor of residuals with loadings with opposite valences. Thus, the authors considered that a multidimensional approach would be the most appropriate. However, instead of using the MCMLM model, the authors considered modeling the positive and negative items in independent clusters and identified a correlation of 0.14 among the latent factors (Santiago et al., 2019). Among our results, the identified correlations present a similar effect size when item 8 is included. However, after removing item 8, the correlations among latent factors were practically null. Another argument supporting the preference of the multidimensional model over the unidimensional one is found in the studies of Nielsen et al. (2016), who identified that the 10-item version of the PSS did not appropriately fit a unidimensional model, which derived in applying a methodology similar to that of Santiago et al. (2019) to contrast multidimensionality. In this way, both studies are previous evidence that the unidimensional structure is not the most appropriate for both versions of the scale and that a multidimensional strategy would be the best option.

### Reliability

Next, the internal consistency by omega coefficient represented for the positive factor (+) ω = 0.919 and for the negative factor (−) ω = 0.859, being these measures superior to the study of Reyna et al. (2019). In turn, the internal consistency through Cronbach’s alpha coefficient reported values > 0.80 for the positive and negative factor, in agreement with what was developed by Cohen et al. (1983), Remor (2006), Lesage et al. (2012), Lee et al. (2015), Pedrero-Pérez et al. (2015), Larzabal-Fernandez and Ramos-Noboa (2019), and Brito Ortíz et al. (2019).

### Public health implications

Based on the results of the confirmatory factor analysis and the Rasch method, a model of 12 items grouped into two orthogonal factors was obtained, so that the effect of one factor does not affect the estimate of the other, so each one provides different information of the construct (Ferrando and Anguiano-Carrasco, 2010) generating a robust product that allows comparing the factors efficiently. Consequently, each scale can be used independently in broad disciplinary fields: health, education, organizational and social community.

## Limitations

Non-probability sampling was one of the main limitations of the present study, whose data collection was carried out in the context of a health emergency, a situation that did not allow formal planning of the selection of participants. Furthermore, we only tested validity evidence based on internal structure and did not address concurrent validity which could have demonstrated insights on the relationships between the PSS scores and other measures previously demonstrated in the literature. In addition, as the sample was collected during the COVID-19 pandemic, other relevant control variables could have been considered in the study.

## Conclusion

Finally, the study contributes to the analysis of the psychometric properties of the PSS with a short version of 12 items in the general population, based on item response theory (Rash Analysis), CFA and invariance according to gender. The uncorrelated factors model offered the best fit by omitting item 8. The confirmation of two differentiated factors as a one-dimensional phenomenon suggests the presence of an alternative measurement model for diagnostic or intervention purposes. The finding of two 12-item stress measurement models is a contribution to psychometrics in clinical contexts as a result of the impact of the state of emergency, benefiting a large sector of the population.

## Data availability statement

The original contributions presented in the study are included in the article/Supplementary material, further inquiries can be directed to the corresponding author.

## Ethics statement

The studies involving human participants were reviewed and approved by Research Ethics Committee, School of Psychology of the Cesar Vallejo University. The patients/participants provided their written informed consent to participate in this study.

## Author contributions

AB-C: conception and design of the study, drafting of the manuscript, statistical analysis, and critical review. MS-C: conception and design of the study, drafting of the manuscript, and statistical analysis. SA: conception and design, data collection, and manuscript writing. AZ-L: drafting of the manuscript, statistical analysis, and critical review. All authors contributed to the article and approved the submitted version.

## Conflict of interest

The authors declare that the research was conducted in the absence of any commercial or financial relationships that could be construed as a potential conflict of interest.

## Publisher’s note

All claims expressed in this article are solely those of the authors and do not necessarily represent those of their affiliated organizations, or those of the publisher, the editors and the reviewers. Any product that may be evaluated in this article, or claim that may be made by its manufacturer, is not guaranteed or endorsed by the publisher.

## References

[ref1] Adams R. J. Wu M. (2007). The mixed-coefficients multinomial Logit model: a generalized form of the Rasch model. In Davier M. von Carstensen C. H. (Eds.), Multivariate and mixture distribution Rasch models: Extensions and applications (57-76). Springer: New York.

[ref2] Andrich D. (1978). A rating formulation for ordered response categories. Psychometrika 43, 561–573. doi: 10.1007/BF02293814

[ref3] Andrich D. Marais I. (2019). A course in Rasch measurement theory: Measuring in the educational, social and health sciences. Singapore: Springer.

[ref4] Baik S. H. Fox R. S. Mills S. D. Roesch S. C. Sadler G. R. Klonoff E. A. . (2019). Reliability and validity of the perceived stress Scale-10 in Hispanic Americans with English or Spanish language preference. J. Health Psychol. 24, 628–639. doi: 10.1177/1359105316684938, PMID: 28810432 PMC6261792

[ref5] Baltar F. Gorjup M. T. (2014). Muestreo mixto online: Una aplicación en poblaciones ocultas. Intangible Capital 8, 123–149. doi: 10.3926/ic.294

[ref6] Barbosa-Leiker C. Kostick M. Lei M. McPherson S. Roper V. Hoekstra T. . (2013). Measurement invariance of the perceived stress scale and latent mean differences across gender and time. Stress. Health 29, 253–260. doi: 10.1002/smi.2463, PMID: 23027679

[ref7] Barraza F. Arancibia M. Madrid E. Papuzinski C. (2019). Conceptos generales en bioestadística y epidemiología clínica: error aleatorio y error sistemático. Medwave 19:e7687. doi: 10.5867/medwave.2019.07.7687, PMID: 31584929

[ref8] Barrio J. A. García M. R. Ruiz I. Arce A. (2006). El estrés como respuesta. Int. J. Develop. Educ. Psychol. 1, 37–48.

[ref9] Benítez A. Danello Koch S. Noel G. (2013). Validación de la escala de estrés percibido (EEP-13) en una muestra de funcionarios públicos venezolanos. Anales de La Universidad Metropolitana 13, 117–133.

[ref10] Bentler P. M. Y. Bonet D. G. (1980). Significance tests and goodness of fit in the analysis of covariance structures. Psychol. Bull. 88, 588–606. doi: 10.1037/0033-2909.88.3.588

[ref11] Bond T. G. Yan Z. Heene M. (2021). Applying the Rasch model: Fundamental measurement in the human sciences. 4th Edn. New York, NY: Routledge.

[ref12] Brito Ortíz J. F. Nava Gómez M. E. Juárez García A. (2019). Escala de estrés percibido en estudiantes de odontología, enfermería y psicología: validez de constructo. Revista Conciencia EPG 4, 42–54. doi: 10.32654/CONCIENCIAEPG.4-2.4

[ref13] Brown T. A. (2015). Confirmatory Factor Analysis 2nd Edn. New York, NY: The Guilford Press.

[ref14] Byrne M. (2008). Testing for multigroup equivalence of a measuring instrument: a walk through the process. Psicothema 20, 872–882. 18940097

[ref15] Campo-AriasA. Pedrozo-CortésM. J. Pedrozo-PupoJ. C. (2020). Carta al Editor: Escala de estrés percibido relacionado con la pandemia de COVID-19: una exploración del desempeño psicométrico en línea. Revista Colombiana de Psiquiatría 49, 229–230. doi: 10.1016/j.rcp.2020.05.005, PMID: 33328014PMC7366950

[ref16] Chen F. F. (2007). Sensitivity of goodness of fit indexes to lack of measurement invariance. Struct. Equ. Modeling 14, 464–504. doi: 10.1080/10705510701301834

[ref17] Ching-Yun Y. (2002). Evaluating cutoff criteria of model fit indices for latent variable models with binary and continuous outcomes. [Doctoral dissertation, University of California]. Available at: http://www.statmodel.com/download/Yudissertation.pdf

[ref18] Cohen S. Kamarck T. Mermelstein R. (1983). A global measure of perceived stress. Journal of health and social behavior. J. Health Soc. Behav. 24, 385–396. 6668417

[ref19] Cohen S. Williamson G. (1988). Perceived stress in a probability sample of the United States. Soc Psychol Health 13, 31–67.

[ref20] Dao-Tran T. H. Anderson D. Seib C. (2017). The Vietnamese version of the perceived stress scale (PSS-10): translation equivalence and psychometric properties among older women. BMC Psychiatry 17, 53–57. doi: 10.1186/s12888-017-1221-6, PMID: 28166754 PMC5295219

[ref22] DiStefano C. Morgan G. B. (2014). A comparison of diagonal weighted least squares robust estimation techniques for ordinal data. Struct. Equ. Modeling 21, 425–438. doi: 10.1080/10705511.2014.915373

[ref23] Domínguez S. (2014). ¿Matrices Policóricas/tetracóricas o Matrices Pearson? Un estudio metodológico. Revista Argentina de Ciencias del Comportamiento 6, 39–48.

[ref24] Dominguez-Lara S. Merino-Soto C. Torres-Villalobos G. (2022). Structural analysis and reliability of the perceived stress scale in nursing professionals from Peru. Enfermería Clínica (English Edition) 32, 152–160. doi: 10.1016/j.enfcle.2022.01.001, PMID: 35568353

[ref25] Dominguez-lara S. A. Rodriguez A. (2017). Índices estadíscos de modelos bifactor. Interacciones 3, 59–65. doi: 10.24016/2017.v3n2.51

[ref26] Dunn T. J. Baguley T. Brunsden V. (2014). From alpha to omega: a practical solution to the pervasive problem of internal consistency estimation. Br. J. Psychol. 105, 399–412. doi: 10.1111/bjop.12046, PMID: 24844115

[ref27] Escobedo Portillo M. T. Hernández Gómez J. A. Estebané Ortega V. Martínez Moreno G. (2016). Modelos de Ecuaciones Estructurales: Características, Fases, Construcción, Aplicación y Resultados structural equation modeling: features, phases, construction, implementation and results. Revista Ciencia y Trabajo 18, 16–22. doi: 10.4067/S0718-24492016000100004

[ref28] Estrada Araoz E. G. Roque M. M. Ramos N. A. G. Uchasara H. J. M. En Educación D. Zuloaga Araoz M. C. . (2021). Estrés académico en estudiantes universitarios peruanos en tiempos de la pandemia del COVID-19. AVFT 40, 88–93. doi: 10.5281/zenodo.4675923

[ref01] Ferrando P. J. Anguiano-Carrasco C. (2010). El análisis factorial como técnica de investigación en psicología. Papeles Del Psicólogo 31, 18–33.

[ref29] Fernández de Larrea-Baz N. Morant-Ginestar C. Catalá-López F. Gènova-Maleras R. Álvarez-Martín E. (2015). Disability-adjusted life years lost to ischemic heart disease in Spain. Rev. Esp. Cardiol. 68, 968–975. doi: 10.1016/j.rec.2014.11.024, PMID: 25887346

[ref30] Finney S. J. DiStefano C. (2013). “Non-normal and categorical data in structural equation modeling” in Structural equation modeling: A second course. eds. Hancock G. R. Mueller R. O.. 2nd ed (Charlotte, NC: Information Age Publishing), 439–492.

[ref31] Flora D. B. (2020). Your coefficient alpha is probably wrong, but which coefficient omega is right? A tutorial on using R to obtain better reliability estimates. Adv. Methods Pract. Psychol. Sci. 3, 484–501. doi: 10.1177/2515245920951747

[ref32] Forero C. G. Maydeu-Olivares A. Gallardo-Pujol D. (2009). Factor analysis with ordinal indicators: a Monte Carlo study comparing DWLS and ULS estimation. Struct. Equ. Model. Multidiscip. J. 16, 625–641. doi: 10.1080/10705510903203573

[ref33] Gelabert E. García-Esteve L. Martín-Santos R. Gutiérrez F. Torres A. Susana S. (2011). Psychometric properties of the frost multidimensional perfectionism scale in Spanish children and adolescents. Assessment 26, 445–464. doi: 10.1177/1073191117740204, PMID: 29117710

[ref34] Guzmán-Yacaman J. E. Reyes-Bossio M. (2018). Adaptation of the global perceived stress scale in college peruvian students. Revista de Psicologia (Peru) 36, 719–750. doi: 10.18800/psico.201802.012

[ref35] Hair J. F. Black W. C. Babin B. J. (2009). Multivariate data analysis: A global perspective. 7th Edn. Upper Saddle River: Prentice Hall.

[ref36] Hoffmann A. F. Stover J. B. (2013). Correlaciones Policóricas Y Tetracóricas En Estudios Factoriales Exploratorios Y Confirmatorios. Ciencias Psicológicas VII, 151–164. doi: 10.22235/cp.v7i1.1057

[ref37] Hu L.-t. Bentler P. M. (1998). Fit indices in covariance structure modeling: sensitivity to underparameterized model misspecification. Psychol. Methods 3, 424–453. doi: 10.1037//1082-989x.3.4.424

[ref38] Hu L.-t. Bentler P. M. (1999). Cutoff criteria for fit indexes in covariance structure analysis: conventional criteria versus new alternatives. Struct. Equ. Modeling 6, 1–55. doi: 10.1080/10705519909540118

[ref39] Huang F. Wang H. Wang Z. Zhang J. Du W. Su C. . (2020). Psychometric properties of the perceived stress scale in a community ample of Chinese. BMC Psychiatry 20, 1–7. doi: 10.1186/s12888-020-02520-4, PMID: 32197589 PMC7082906

[ref40] Hyde J. S. (2005). The gender similarities hypothesis. Am. Psychol. 60, 581–592. doi: 10.1037/0003-066X.60.6.581 16173891

[ref41] Jasis M. Guendelman S. (1993). Maquiladoras y mujeres fronterizas: beneficio o daño a la salud obrera? Salud Publica Mex. 35, 620–629. 8128301

[ref42] Jerez Ríos M. P. Madero-Cabib I. (2021). Trayectorias de estrés familiar y laboral y su asociación con accidentes cerebrovasculares. Rev. Saude Publica 55:101. doi: 10.11606/s1518-8787.20210550033253 34910029 PMC8621564

[ref43] Johnson T. P. (2014). Snowball sampling: introduction Wiley StatsRef, 12–14.

[ref44] Jöreskog K. G. (1994). On the estimation of polychoric correlations and their asymptotic covariance matrix. Psychometrika 59, 381–389. doi: 10.1007/BF02296131

[ref45] Juárez-García A. Monroy-Castillo A. Gómez E. N. Juárez-García A. Nava-Gómez M. E. (2021). ¿Es la escala de estrés percibido (PSS) Undimensional e invariante? Un análisis bifactorial en adultos mexicanos. *Current Psychol*. 42, 7252–7266. doi: 10.1007/s12144-021-02067-x

[ref46] Kline R. B. (2005). Principles and practice of structural equation modeling (2nd Edn.). Nueva York, NY: Guilford.

[ref47] Kline R. B. (2011). Principles and practice of structural equation modeling. New York: Guilford Press.

[ref48] Lai K. (2020). Fit difference between nonnested models given categorical data: measures and estimation. Struct. Equ. Modeling 28, 99–120. doi: 10.1080/10705511.2020.1763802

[ref49] Lamprianou I. (2020). Applying the Rasch model in the social sciences using R and BlueSky statistics. New York, NY: Routledge.

[ref50] Larzabal-Fernandez A. Ramos-Noboa M. I. (2019). Propiedades psicométricas de la Escala de Estrés Percibido (PSS-14) en estudiantes de bachillerato de la provincia de Tungurahua (Ecuador). Ajayu 17, 269–282.

[ref51] Lee E. H. Chung B. Y. Suh C. H. Jung J. Y. (2015). Korean versions of the perceived stress scale (PSS-14, 10 and 4): psychometric evaluation in patients with chronic disease. Scand. J. Caring Sci. 29, 183–192. doi: 10.1111/scs.12131, PMID: 24660854

[ref52] León Regal M. García Álvarez Y. Álvarez Hernández R. Morales Pérez C. Regal Cuesta V. González León H. (2018). Influencia del estrés psicológico y la actividad física moderada en la reactividad cardiovascular. Revista Finlay 8, 224–233.

[ref53] Lesage F. X. Berjot S. Deschamps F. (2012). Psychometric properties of the french versions of the perceived stress scale. Int. J. Occup. Med. Environ. Health 25, 178–184. doi: 10.2478/S13382-012-0024-8, PMID: 22528542

[ref54] Li C. H. (2016). Confirmatory factor analysis with ordinal data: comparing robust maximum likelihood and diagonally weighted least squares. Behav. Res. Methods 48, 936–949. doi: 10.3758/s13428-015-0619-7, PMID: 26174714

[ref55] Lloret S. Ferreres A. Tomás A. H. (2017). El análisis factorial exploratorio de los ítems: Análisis guiado según los datos empíricos y el software. Anales de Psicologia 33, 417–432. doi: 10.6018/analesps.33.2.270211

[ref56] Lloret-Segura S. Ferreres-Traver A. Hernández-Baeza A. Tomás-Marco I. (2014). El análisis factorial exploratorio de los ítems: una guía práctica, revisada y actualizada Introducción Determinación de la adecuación del Análisis. Anales De Psicología 30, 1151–1169. doi: 10.6018/analesps.30.3.199361

[ref57] López V. Millares Cao C. Fajardo R. Lera L. (1995). Características psicológicas de los enfermos de vitiligo. Revista Cubana de Psicologia 12, 245–253.

[ref58] Maroufizadeh S. Foroudifard F. Navid B. Ezabadi Z. Sobati B. Omani-Samani R. (2018). The perceived stress scale (PSS-10) in women experiencing infertility: a reliability and validity study. Middle East Fertility Soc J 23, 456–459. doi: 10.1016/j.mefs.2018.02.003

[ref59] Martin I. M. (2007). Estrés Académico en Estudiantes Universitarios. Apuntes de Psicología 14, 42–47. doi: 10.37843/rted.v14i2.330

[ref60] Martínez C. M. Sepúlveda M. A. R. (2012). Introducción al análisis factorial exploratorio. Revista Colombiana de Psiquiatría 41, 197–207. doi: 10.1016/s0034-7450(14)60077-9, PMID: 26573478

[ref61] Maydeu-Olivares A. (2013). Goodness-of-fit assessment of item response theory models. Measurement Interdisciplin Res Perspect 11, 71–101. doi: 10.1080/15366367.2013.831680

[ref02] Medvedev O. N. Krägeloh C. U. Hill E. M. Billington R. Siegert R. J. Webster C. S. . (2019). Rasch analysis of the Perceived Stress Scale: Transformation from an ordinal to a linear measure. J. Health Psychol. 24, 1070–1081. doi: 10.1177/1359105316689603, PMID: 28810395

[ref03] Moral de la Rubia J. Cázares De León F. (2014). Validación de la Escala de Estrés percibido (PSS-14) en la población de dentistas colegiados de Monterrey. Ansiedad y Estrés 20, 193–209., PMID: 30664405

[ref62] Moreno R. Martínez R. J. Muñiz J. (2006). New guidelines for developing multiple-choice items. Methodology 2, 65–72. doi: 10.1027/1614-2241.2.2.65

[ref63] Muñiz J. Fonseca-Pedrero E. (2019). Ten steps for test development. Psicothema 31, 7–16. doi: 10.7334/psicothema2018.291, PMID: 30664405

[ref64] Nielsen M. G. Ørnbøl E. Vestergaard M. Bech P. Larsen F. B. Lasgaard M. . (2016). The construct validity of the perceived stress scale. J. Psychosom. Res. 84, 22–30. doi: 10.1016/j.jpsychores.2016.03.009 27095155

[ref65] Organizacion Mundial de la Salud . (2020). Los Servicios de Salud mental se están viendo perturbados por la COVID-19 en la mayoría de los países, según un estudio de la OMS. Available at: https://www.who.int/es/news/item/05-10-2020-covid-19-disrupting-mental-health-services-in-most-countries-who-survey

[ref66] Organización Panamericana de la Salud (2022). La pandemia por COVID-19 provoca un aumento del 25% en la prevalencia de la ansiedad y la depresión en todo el mundo. Available at: https://www.paho.org/es/noticias/2-3-2022-pandemia-por-covid-19-provoca-aumento-25-prevalencia-ansiedad-depresion-todo

[ref04] Palomino-Oré C. Huarcaya-Victoria J. (2020). Trastornos por estrés debido a la cuarentena durante la pandemia por la COVID-19. Horiz. Med. 20:e1218. doi: 10.24265/horizmed.2020.v20n4.10

[ref67] Pedrero-Pérez E. J. Ruiz-Sánchez de León J. M. Lozoya-Delgado P. Rojo-Mota G. Llanero-Luque M. Puerta-García C. (2015). La “escala de estrÉs percibido”: estudio psicométrico sin estricciones en población no clÍnica y adictos a sustancias en tratamiento. Behav Psychol 23, 305–324.

[ref68] Pérez E. Medrano L. (2010). Análisis Factorial Exploratorio: Bases Conceptuales y Metodológicas Artículo de Revisión. TEST 2, 58–66.

[ref69] Puentes Martínez L. Beatriz A. Rábago D. (2019). Reliability and construct validity of the perceived stress scale in medical students. Rev. Ciencias Médicas 23, 373–379.

[ref70] Reise S. P. Scheines R. Widaman K. F. Haviland M. G. (2013). Multidimensionality and structural coefficient Bias in structural equation modeling: a Bifactor perspective. Educ. Psychol. Meas. 73, 5–26. doi: 10.1177/0013164412449831

[ref71] Remor E. (2006). Propiedades psicométricas de una versión española europea de la Escala de Estrés Percibido (PSS). Redalyc 9, 86–93.

[ref72] Remor E. Carrobles J. A. (2001). Versión española de la escala de estrés percibido (PSS-14): Estudio psicométrico en una muestra VIH+. Ansiedad Estrés 7, 195–201.

[ref73] Revelle W. (2023). Psych: Procedures for Psychological, Psychometric, and Personality Research. Northwestern University, Evanston, Illinois. R package version 2.3.3. Available at: https://CRAN.R-project.org/package=psych

[ref74] Reyna C. Mola D. J. Correa P. S. (2019). Escala de Estrés Percibido: análisis psicométrico desde la TCT y la TRI. Psychometr. Anal. 25, 138–147. doi: 10.1016/j.anyes.2019.04.003

[ref75] Roberti J. W. Harrington L. N. Storch E. A. (2006). Further psychometric support for the 10-item version of the perceived stress scale. J. Coll. Couns. 9, 135–147. doi: 10.1002/j.2161-1882.2006.tb00100.x

[ref76] Rodrigo-Comino J. Taguas E. V. Seeger M. K. Ries J. B. (2019). Evaluación de los procesos superficiales de escorrentía en cárcavas originadas en olivares convencionales. Un apartado a tener en cuenta en la planificación territorial. Revista de Geografía Norte Grande 248, 229–248. doi: 10.4067/s0718-34022019000300229

[ref77] Rodriguez A. Reise S. P. Haviland M. G. (2016). Applying bifactor statistical indices in the evaluation of psychological measures: Correction. J. Personality Assess. 98:444. doi: 10.1080/00223891.2015.1117928 26514921

[ref78] Rosseel Y. (2012). Lavaan: an R package for structural equation modeling. J. Stat. Softw. 48, 1–36. doi: 10.18637/jss.v048.i02

[ref79] Sánchez P. I. G. (2009). Principios básicos de bioética. Revista Peruana De Ginecología y Obstetricia 55, 1–12.

[ref80] Sandoval-Reyes J. Idrovo-Carlier S. Duque-Oliva E. J. (2021). Remote work, work stress, and 628 work–life during pandemic times: a Latin America situation. Int J Environmental Res Public Health 18:7069. doi: 10.3390/ijerph18137069, PMID: 34281007 PMC8297005

[ref81] Santiago P. H. R. Roberts R. Smithers L. G. Jamieson L. (2019). Stress beyond coping? A Rasch analysis of the perceived stress scale (PSS-14) in an aboriginal population. PLoS One 14, e0216333–e0216324. doi: 10.1371/journal.pone.0216333, PMID: 31050685 PMC6499425

[ref82] Sijtsma K. (2009). On the use, the misuse, and the very limited usefulness of Cronbach’s alpha. Psychometrika 74, 107–120. doi: 10.1007/s11336-008-9101-0, PMID: 20037639 PMC2792363

[ref83] Steiner M. Grieder S. (2020). EFAtools: an R package with fast and flexible implementations of exploratory factor analysis tools. J Open Source Softw 5:2521. doi: 10.21105/joss.02521

[ref84] Sun Y. Gao L. Kan Y. Shi B. X. (2019). The perceived stress Scale-10 (PSS-10) is reliable and has construct validity in Chinese patients with systemic lupus erythematosus. Lupus 28, 149–155. doi: 10.1177/0961203318815595, PMID: 30518288

[ref85] Ten Berge J. M. F. Sočan G. (2004). The greatest lower bound to the reliability of a test and the hypothesis of unidimensionality. Psychometrika 69, 613–625. doi: 10.1007/BF02289858

[ref86] Tomas J. Galiana L. Hontangas P. Oliver A. Sancho P. (2013). Evidencia acumulada sobre los efectos de método asociados a ítems invertidos La práctica y recomendación de invertir ítems al medir constructos psicológicos mediante escalas ha estado presente casi desde siempre en la medición psicológica y educativa De. Psicológica 34, 365–381.

[ref87] Tomás J. M. Requena P. S. Germes A. O. Llinares L. G. Moral J. C. M. (2012). Efectos de método asociados a ítems invertidos vs. ítems en negativo. Revista Mexicana de Psicologia 29, 105–115.

[ref88] Trujillo H. M. González-Cabrera J. M. (2007). Propiedades psicométricas de la versión española de la Escala de Estrés Percibido (EEP). Psicol. Conduct. 15, 457–477.

[ref89] Ventura-León J. L. (2018). ¿Es el final del alfa de Cronbach? Adicciones 31, 80–81. doi: 10.20882/adicciones.1037, PMID: 30059590

[ref90] Wright B. D. (1993). Thinking with raw scores. Rasch Measurement Transact 7, 299–300.

[ref91] Wright B. D. Linacre J. M. (1994). Reasonable mean-square fit values. Rasch Measurement Transact 8, 370–371.

[ref92] Xia Y. Yang Y. (2019). RMSEA, CFI, and TLI in structural equation modeling with ordered categorical data: the story they tell depends on the estimation methods. Behav. Res. Methods 51, 409–428. doi: 10.3758/s13428-018-1055-2, PMID: 29869222

[ref93] Yokokura A. V. C. Moura Da Silva A. A. Del-Ben C. M. De Figueiredo F. P. Barbieri M. A. Bettiol H. (2017). Perceived stress scale: confirmatory factor analysis of the PSS14 and PSS10 versions in two samples of pregnant women from the BRISA cohort. Cad. Saude Publica 33, e00184615–e00184613. doi: 10.1590/0102-311X00184615, PMID: 29267695

[ref94] Zinbarg R. E. Yovel I. Revelle W. McDonald R. P. (2006). Estimating generalizability to a latent variable common to all of a scale’s indicators: a comparison of estimators for ωh. Appl. Psychol. Measur. 30, 121–144. doi: 10.1177/0146621605278814

